# Death While Unconscious from Ether

**Published:** 1875-12

**Authors:** 


*Death while Unconscious from Ether.*—In New York, on Saturday, an inquest was held on the body of James H. Hallock, who died from the effects of ether administered to him while undergoing an operation on his jaw. Dr. W. T. Helmuth testified that he performed the operation on Mr. Hallock, and that the ether was given to him, an assistant physician constantly feeling the patient's pulse. The Doctor had extracted four teeth, when he noticed that the face of the patient had become blue. Artificial respiration and the galvanic battery were instantly resorted to, but without avail. Witness had often given six or eight times the quantity of ether during an operation without injurious effects. It was also testified that Hallock had been in good general health, but for several weeks had been suffering from an ulcerated jaw.

